# Analysis of factors influencing the distribution of 131-I in combined treatment of Licartin with transcatheter arterial chemoembolization in primary hepatic carcinoma

**DOI:** 10.3389/fonc.2022.993948

**Published:** 2023-03-13

**Authors:** Ming Tang, Wen-Liang Li, Jia-Yu Li, Juan Lv, Fu-Kun Chen, Jia-Lun Zhu, Peng-Jie Liu

**Affiliations:** ^1^ Department of Pathology, The First People’s Hospital of Yunnan Province, The Affiliated Hospital of Kunming University of Science and Technology, Kunming, China; ^2^ Department of gynecology, The Cancer Hospital of Yunnan Province, The Third Affiliated Hospital of Kunming Medical University, Kunming, China; ^3^ Department of Nuclear Medical, The First People’s Hospital of Zhaotong City, Zhaotong, China; ^4^ Department of Nuclear Medical, The Cancer Hospital of Yunnan Province, The Third Affiliated Hospital of Kunming Medical University, Kunming, China

**Keywords:** Hepatocellular carcinoma, transcatheter arterial chemoembolization, Licartin (^131^I Metuximab), combined treatment, 131-I

## Abstract

**Objective:**

To analyze the factors influencing the distribution of 131-I in the liver of patients with advanced hepatic carcinoma treated with the combination of Licartin (^131^I Metuximab) and transcatheter arterial chemoembolization (TACE). This study provides a reference and basis for the clinic on how to choose the best time for the treatment of Licartin and how to reduce other possible factors affecting the role of Licartin.

**Methods:**

Data from 41 patients with advanced hepatic carcinoma treated with the combination of Licartin and TACE in the Interventional Department of our hospital from March 2014 to December 2020 were collected. This included general characteristics, history of open and interventional surgery, interval between the last interventional surgery and the Licartin treatment, selected arteries in the Licartin perfusion, and 131-I distribution in the liver. Regression analysis was conducted to investigate the factors affecting the distribution of ^131^I in the liver.

**Results:**

In 14 cases (34.1%), 131-I was evenly distributed in the liver, and there was no correlation between the cause of even distribution with age(OR=0.961, P = 0.939), previous open surgery history(OR=3.547,P= 0.128), previous history of interventional therapy(OR=0.140,P = 0.072), the interval between the last interventional surgery and the Licartin treatment(OR=0.858,P = 0.883), or the choice of the perfusion artery in the Licartin treatment (OR=1.489,P = 0.419). In 14 cases (34.1%), there was higher aggregation in the tumor than in the normal liver, which was related to previous interventional surgery (OR=7.443,P = 0.043). In 13 cases (31.7%), there was lower aggregation in the tumor than in the normal liver, which was related to the selected vessels in the Licartin perfusion (OR=0.23,P = 0.013).

**Conclusion:**

The effective aggregation of 131-I in the liver, even in tumors, the previous history of TACE, and the choice of vessels in the Licartin infusion might be the factors influencing the distribution of 131-I in the liver during hepatic artery infusion of Licartin in combination with TACE therapy.

## Introduction

Hepatocellular carcinoma (HCC) has a high mortality, which is second only to lung cancer in China (1). In the early stage, HCC has no obvious symptoms, and it is usually diagnosed in the middle or late stage when there is usually no opportunity for surgery. Predicting postoperative recurrence and treatment for HCC is difficult (2, 3). Transcatheter arterial chemoembolization (TACE) is an important treatment for patients with HCC (4), and it can control tumor growth to a certain extent and delay the progression of the disease. Licartin (^131^I Metuximab) is the first radioimmune agent independently developed in China to treat HCC. Transhepatic arterial infusion of Licartin combined with TACE can improve local efficacy compared with TACE alone (5), and it is an effective method for treating unresectable advanced or recurrent HCC (6–8). It has a good anti-recurrence effect and significantly improves the 5-year survival rate in advanced hepatic carcinoma (9). Due to the specificity of rituximab, the distribution of 131I should be mainly in the liver tumor tissue after treatment with Licartin, but there are cases of uniform distribution and increased or decreased distribution in the tumor area in clinic. Since Licartin is injected into the liver through intervention, the relevant factors affecting the vascular structure of the liver may be the key factors affecting the distribution of Licartin. Among these factors, the history of previous open surgery and interventional surgery are the two most important factors affecting the vascular structure, and the interval between the last interventional surgery and the treatment with Licartin is one of the factors affecting the vascular regeneration. Therefore, this study aimed to find out the reasons for the existence of multiple distribution forms of Licartin in the liver by analyzing the patient’s age, previous open surgery history, previous interventional treatment history, the interval between the last interventional operation and the treatment of Licartin, and the perfusion artery selected during the treatment of riccatine.

## Materials and methods

### Study subjects

From March 2014 to December 2020, 41 patients with advanced HCC who received a sequential hepatic arterial infusion of Licartin combined with TACE in the Interventional Department of our hospital were enrolled in the study. The inclusion criteria were as following. Patient’s child-Pugh grade was A. Patients who could not tolerate surgery or had postoperative recurrence. Patients had diseases recurrence after TACE treatment.

### Procedure

The whole operation was performed under the DSA machine, with the right femoral artery or right radial artery approach. Tumor feeding vessels were selected as the first choice, followed by proper hepatic artery and common hepatic artery. Briefly, patients would be treated with 5mg Metuximab. Oral administration of Lugol’s solution 3 days before Licartin treatment. Took Lugol orally, 0.5ml/time, three times a day for 10 days. The injection of Licartin should be completed within 5-10min. After the injection, 0.9% normal saline was used to flush the intubation to ensure that all drugs enter. The radioactivity used for 131-I was 27.75 MBq/kg. After the intraoperative angiography showed the branch of the left gastric artery, which was the blood supply artery of the tumor, 50mg Calcium levophyllate, 250mg 5FU and 10mg Epirubicin were injected through SP micro tube. After that, Licartin and gelatin sponge particles were injected into the catheter for embolization until the embolization was satisfactory. 131-I whole-body-scan would be performed on the 5th to 7th day after Licartin perfusion. The average time was 6.2 days. The drug distribution was detected by SPECT scanning (SIEMEMS, German). The matrix size was set as 256*1024, and the zoom was set as 1.00. The detect speed was set as 15 cm/min. Then, it would be used to compare with the data of CT and MRI to make sure the distribution of 131-I in tumor and normal tissue.

### Data collection

Data on age, previous open surgery history, previous history of interventional treatment, the interval between the last interventional surgery and the Licartin treatment, the arterial perfusion selected in the Licartin treatment, and the hepatic distribution of 131-I were collected. The statistical assignment was conducted for the six items (**Table 1**). The distribution of 131-I in the liver was analyzed by 131-I whole-body scanning and the tumor/non-tumor (T/NT) ratio of the hepatic tumor to the surrounding normal liver tissue. The results showed that when 0.9 ≤ the T/NT ratio ≤ 1.1, there was no difference in the 131-I distribution between the tumor region and the normal liver tissue. When the T/NT ratio > 1.1, the 131-I distribution in the tumor lesions was higher than in the normal liver. When T/NT < 0.9, the 131-I distribution in the tumor lesion was considered to be lower than in the normal liver tissue.

**Table 1:** Assignment table of variables influencing the distribution of Licartin in the region of liver malignancy.

Variables	Assignment	Variables	Assignment
Age	1>41years old, ≤50 years old;2>50 years old, ≤60 years old; 3>60 years old, ≤69 years old	Increased Licartin distribution in tumor areas	1=yes; 2=no
Previous history of open surgery	1=yes; 2=no	Even Licartin distributed in the tumor area	1= uniform; 2=non-uniform
Previous history of TACE	1=no; 2=yes	Decreased Licartin distribution in the tumor area	1=decrease; 2=increase
Selection of vessels in Licartin perfusion	1=Super selective tumor artery; 2=proper hepatic artery; 3=common hepatic artery	The interval between the last surgery and Licartin perfusion surgery	1 ≤ 2 weeks; 2>2 weeks

### Statistical analysis

The SPSS™ Statistics v25.0 software was used for statistical analysis, and binary logistic regression was conducted. The odds ratio (OR) and 95% confidence interval (95% CI) of each factor were estimated, and the test level was set at α = 0.05, meaning P < 0.05 was considered statistically significant.

## Results

Of the 41 patients, 34 were male and 7 were female. Ages ranged from 41 to 69 years old. Of these, 14 cases were ≤50 years old and 27 cases were >50 years old, and the average age was 55.0 ± 8.9 years old. Regarding previous surgery, 8 patients had previous open surgery only, 14 patients had previous TACE only, 14 patients had previous open surgery and TACE, and 5 patients had neither previous open surgery nor TACE. The interval between the last surgery and combined treatment of Licartin with TACE was ≤2 weeks in 9 patients, including 5 patients who had no surgery, and 32 patients had an interval of >2 weeks. The selection of vessels in the Licartin perfusion were as follows: super-selected tumor vessels in 24 cases, the proper hepatic artery in 9 cases, and the common hepatic artery in 8 cases. The distribution of Licartin in the tumor area was higher distribution in 14 cases (34.1%). The Licartin distribution in the tumor region was even distribution in 14 cases (34.1%). The distribution of Licartin in the tumor area was lower distribution in 13 cases (31.7%).

### Results of binary logistic regression analysis

**Figure 1:**
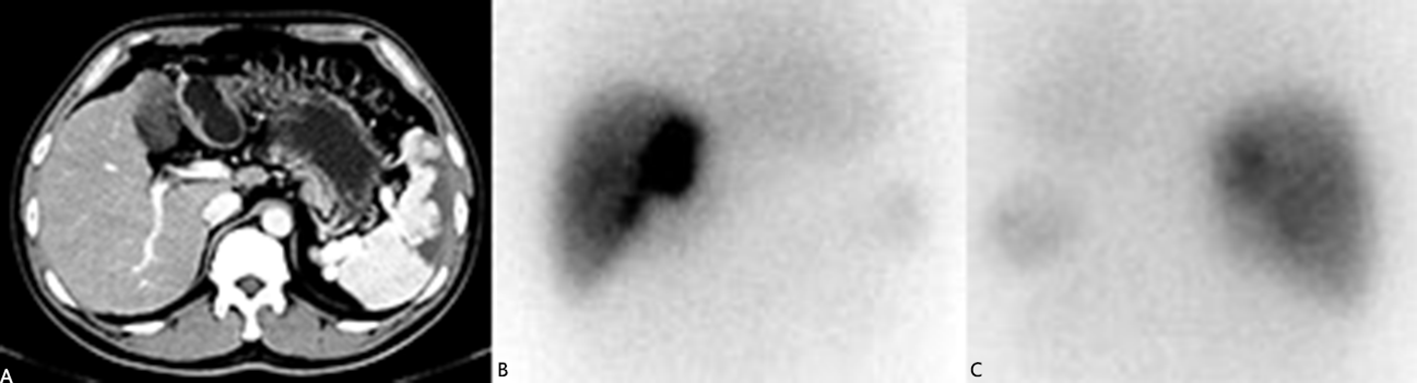
**(A)** The liver computed tomography suggested the postoperative stump recurrence of the right lobe (5.8 × 3.6 cm^2^). High density shadow of spleen could be seen. This patient had splenic artery embolism before due to hypersplenism; **(B)** The anterior image of the hepatic area in 131-I whole-body scanning revealed increased 131-I uptake in recurrent tumors; **(C)** The posterior image of the hepatic area in 131-I whole-body scanning revealed increased 131-I uptake in recurrent tumors.

The Licartin distribution was more concentrated in the tumor area than in the normal liver tissue (**Figure 1**). This distribution pattern was correlated with previous TACE (OR=7.443, P = 0.043). But it was not correlated with age (OR=1.572, P = 0.432), previous open surgery (OR=0.253, P = 0.212), the surgical interval between the last operation and Licartin combined with TACE (OR=1.541, P = 0.653), and selected vessels (OR=9.329, P = 0.061). Specific results are shown in **Table 2**.

**Table 2:** Binary logistic regression analysis of factors resulting in the increased Licartin distribution in hepatic carcinoma area compared with the normal liver tissue.

Factors	B	SE	Wald	*P value*	*OR*	*OR*(*95%CI*)
Age	0.452	0.688	0.432	0.432	1.572	0.408∼6.050
History of open surgery	-1.374	1.101	1.557	0.212	0.253	0.029∼2.191
History of TACE	2.007	0.991	4.106	0.043	7.443	1.068∼51.864
The interval between the last operation and Licartin surgery	0.432	0.962	0.202	0.653	1.541	0.234∼10.152
Blood vessels for Licartin perfusion	2.233	1.192	3.512	0.061	9.329	0.903∼96.423

B: the partial regression coefficient; SE, the standard error; Wald, the value of chi-square test.

### Licartin distribution in the hepatic carcinoma

**Figure 2:**
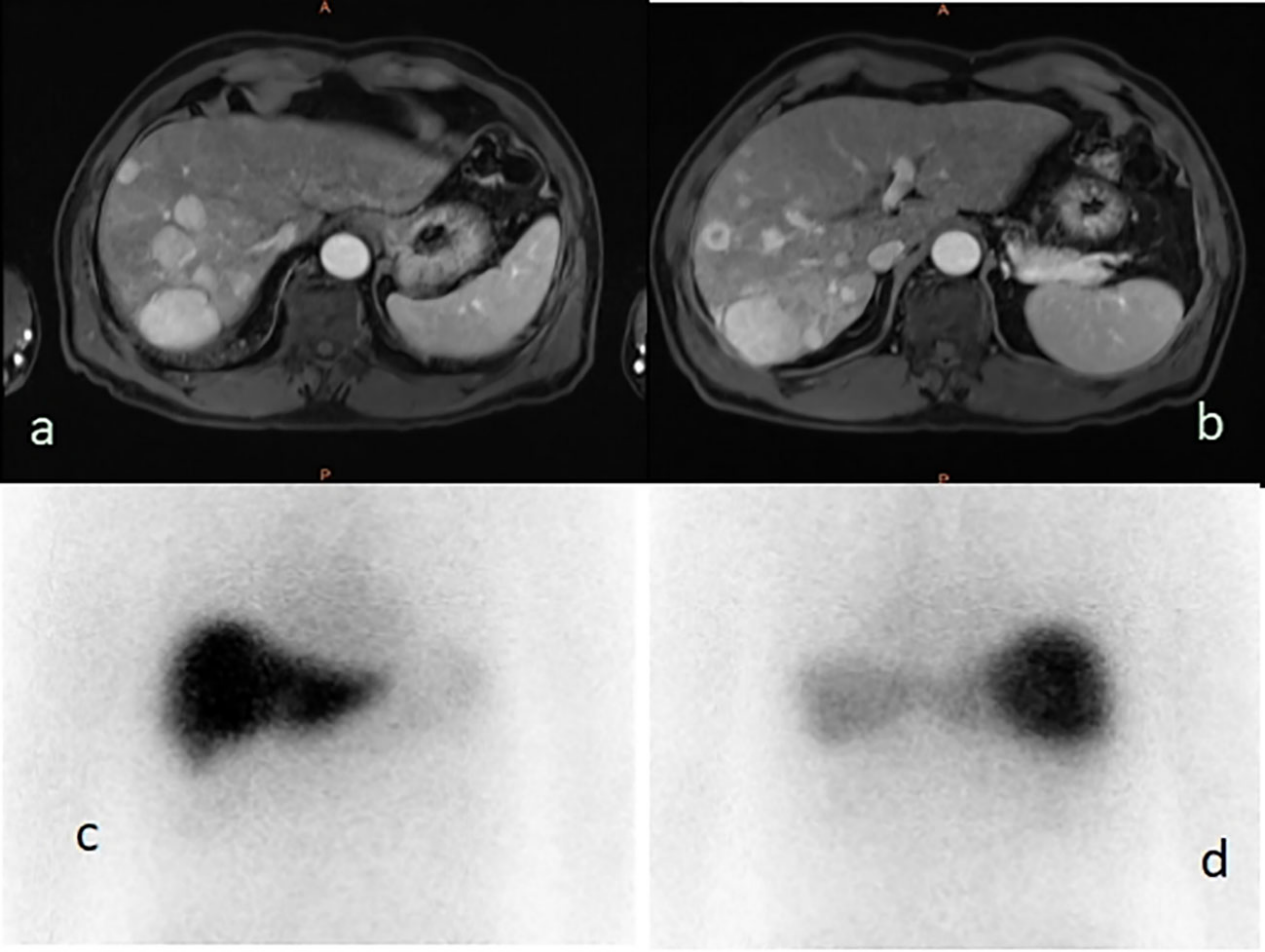
**(A, B)** The liver magnetic resonance imaging demonstrated multiple parenchymal nodules in right lobe of liver (5.0 × 3.8 cm^2^); **(C)** The 131-I whole body scan showed that the right and left lobes of the liver were evenly uptake of 131-I before, and the multiple nodules showed no higher uptake than the surrounding liver tissues; **(D)** The 131-I whole body scan showed that the right lobe of the liver was evenly uptake of 131-I, and the multiple nodules showed no higher uptake than the surrounding liver tissues.

Licartin distribution in the hepatic carcinoma was not significantly different from that in the normal liver tissue (**Figure 2**). This distribution pattern was not correlated with age (OR=0.961,P = 0.939), previous open surgery (OR=3.547,P = 0.128), the previous history of interventional therapy (OR=0.140,P = 0.072), the surgical interval between the last operation and Licartin combined with TACE (OR=0.858,P = 0.883), and selected vessels (OR=1.489,P = 0.419). The details are shown in **Table 3**.

**Table 3:** Binary logistic regression analysis of factors resulting in the even Licartin distribution between the hepatic carcinoma area and to the normal liver tissue.

Factors	B	SE	Wald	*P value*	*OR*	*OR*(*95%CI*)
Age	-0.040	0.520	0.006	0.939	0.961	0.347∼2.665
Open surgery	1.266	0.831	2.320	0.128	3.547	0.695∼18.097
Previous history of TACE	-1.965	1.094	3.229	0.072	0.140	0.016∼1.195
The interval between the last operation and Licartin surgery	-0.153	1.036	0.022	0.883	0.858	0.113∼6.535
Blood vessels for injection	0.398	0.493	0.654	0.419	1.489	0.567∼3.910

### Licartin distribution in the hepatic tumor area

**Figure 3:**
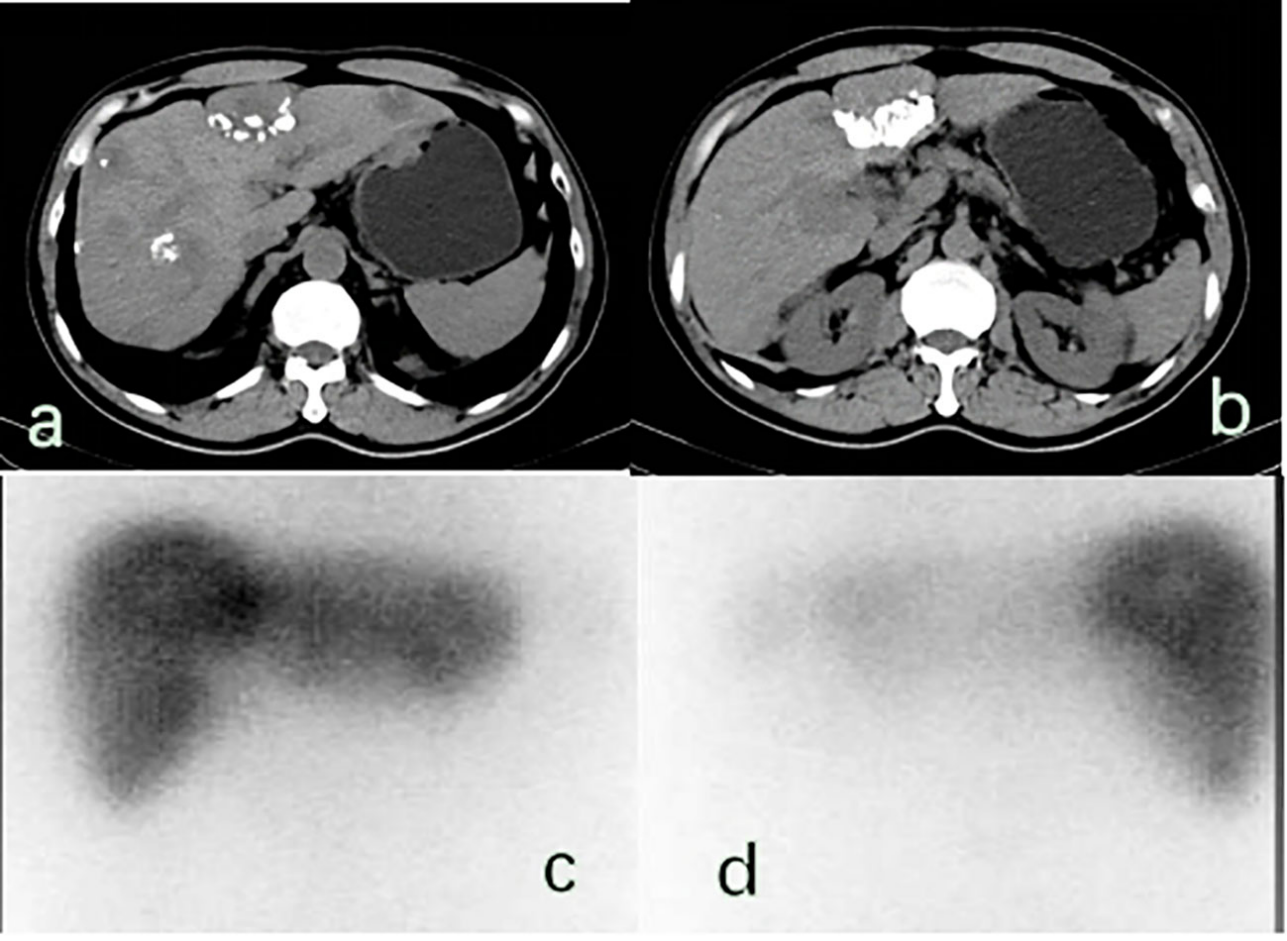
**(A, B)** There were multiple space occupying lesions in the liver with lipiodol deposition, and the largest was located in the left inner lobe of the live (5.7× 4.1 cm^2^).; **(C, D)** 131-I whole-body scan showed that 131-I absorbed by the liver was unevenly distributed in the anterior and posterior positions, and the areas with reduced radioactivity were mostly areas with lipiodol deposition, suggesting that the combination of Licartin and liver tumors was affected by previous TACE operations.

The Licartin distribution in the hepatic tumor area was less than in the normal liver tissue (**Figure 3**). This distribution pattern was not correlated with age (OR=1.043,P = 0.940), previous open surgery (OR=0.528,P = 0.511), the previous history of interventional therapy (OR=0.756,P = 0.786), and the surgical interval between the last interventional therapy and Licartin (OR=1.077,P = 0.947). However, it was correlated with the selected arteries in the Licartin treatment (OR=0.230,P = 0.012). The detailed results are illustrated in **Table 4**.

**Table 4:** Binary logistic regression analysis of factors resulting in the decreased Licartin distribution in hepatic carcinoma area compared with the normal liver tissue.

Factors	B	SE	Wald	*P value*	*OR*	*OR*(*95%CI*)
Age	0.042	0.559	0.006	0.940	1.043	0.349∼3.119
Open surgery	-0.638	0.970	0.432	0.511	0.528	0.079∼3.537
Previous history of TACE	-0.280	1.030	0.074	0.786	0.756	0.100∼5.692
The interval between the last operation and Licartin surgery	-0.074	1.110	0.004	0.947	1.077	0.122∼9.478
Blood vessels for injection	-1.471	0.584	6.342	0.012	0.230	0.073∼0.722

### The univariate analysis of vessel selected

The injecting vessels was an important link for Licartin to enter the liver. Therefore, we subjected vessel selected into univariate analysis (**Table 5**). The results showed that the increased distribution of Licartin in tumor was related to the selected injection artery. The super-selected tumor arteries were the promoting factor for the increased distribution of Licartin in tumor. The selection of super-selected tumor arteries significantly increased the accumulation of Licartin in liver tumors (OR=18.909, P=0.008) (**Table 6**). Besides, it was also the protection factor to avoid the decreased distribution of Licartin in liver tumors (OR=0.100, P=0.004) (**Table 7**). The injection of Licartin from the common hepatic artery was a promoting factor to reduce the distribution of drug in liver tumors (OR=5.208, P=0.048) (**Table 7**). There was no correlation between the distribution of Licartin in the liver and the selection of injection vessels (**Table 8**).

**Table 5:** Distribution analysis of injected arterial variables affecting the regional distribution of Licartin in liver malignant tumors.

Variables	Assignment	Variables	Assignment
Super selective tumor artery	1=yes; 2=no	Increased Licartin distribution in tumor areas	1=yes; 2=no
proper hepatic artery	1=yes; 2=no	Even Licartin distributed in the tumor area	1=uniform; 2=non-uniform
common hepatic artery	1=yes; 2=no	Decreased Licartin distribution in the tumor area	1=decrease; 2=increase

**Table 6:** Binary regression analysis on the relationship between the injection of Licartin into the artery and the increase in the distribution of Licartin.

Factors	B	SE	Wald	*P value*	*OR*	*OR* (*95%CI*)
Super selective tumor artery	0.657	0.329	3.977	0.008	18.909	2.150∼166.275
proper hepatic artery	-1.700	1.120	2.303	0.129	0.183	0.020∼1.641
common hepatic artery	-20.898	14210.4	0.00	0.999	0.000	0.000

**Table 7:** Binary regression analysis of the relationship between the injection of Licartin into arteries and the decrease of the distribution of Licartin.

Factors	B	SE	Wald	*P value*	*OR*	*OR*(*95%CI*)
Super selective tumor artery	-2.303	0.790	8.499	0.004	0.100	0.021∼0.470
proper hepatic artery	1.322	0.785	2.833	0.092	3.750	0.805∼17.477
common hepatic artery	1.650	0.836	3.900	0.048	5.208	1.012∼26.793

**Table 8:** Binary regression analysis between the injection of Riccatin into arteries and the uniform distribution of Licartin.

Factors	B	SE	Wald	*P value*	*OR*	*OR*(*95%CI*)
Super selective tumor artery	-0.087	0.667	0.017	0.896	0.917	0.248∼3.389
proper hepatic artery	-0.047	0.799	0.003	0.954	0.955	0.199∼4.571
common hepatic artery	0.182	0.818	0.050	0.824	1.200	0.241∼5.967

### The adverse effect of Licartin treatment

**Figure 4:**
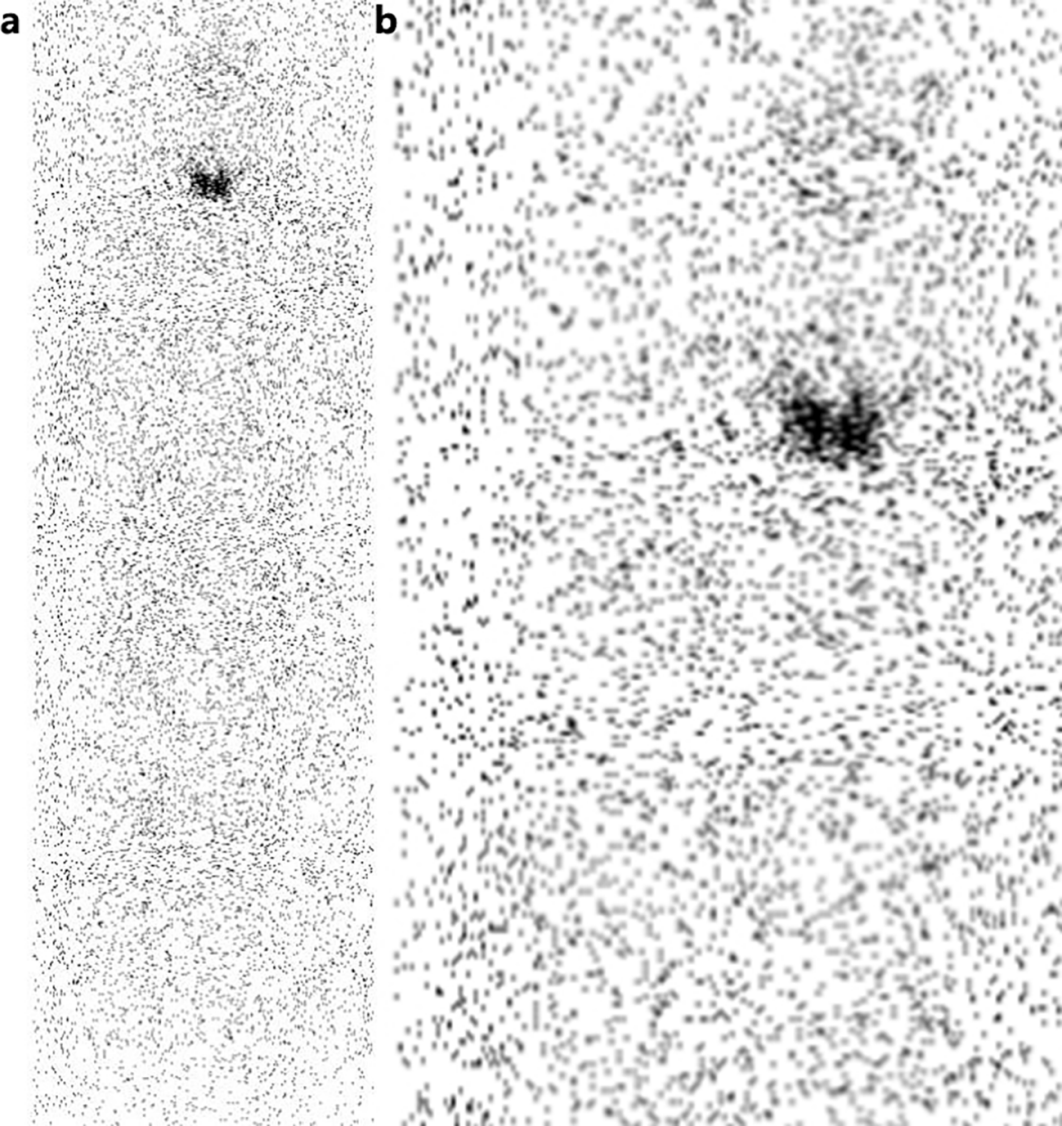
**(A)** Radioactive uptake of thyroid gland can be seen on the whole body 131-I scan 1 month after hepatic artery perfusion of 131-I metoximab combined with TACE. No uptake was observed in liver and other organs. **(B)** Local manifestation of thyroid region.

Generally, the most common adverse effect of TACE combined with Licartin in the treatment of liver cancer patients was the post embolism syndrome caused by TACE, which was mainly manifested as fever, pain, nausea and vomiting. In this study, no patient had shown obvious adverse effect. However, one patient underwent 131-I whole-body scanning one month after treatment, and the results showed that 131-I uptake of thyroid gland was found (**Figure 4**). Meanwhile, thyroid function examination indicated hypothyroidism. Whether this effect on thyroid function was a common phenomenon need further study, and the protective measures for thyroid need to be further strengthened and improved.

## Discussion

Generally, 131-I is adopted to treat differentiated thyroid cancer with the mechanism of high-energy beta rays to kill tumor cells. TACE has been used as the standard treatment globally for patients with intermediate HCC (10), with median survival of 19.4 months in the uncontrolled study (11) and 37 months in RCT (12–15). An institutional experience in the United States reported that compared with TACE alone, 5-year DFS could be improved by 33.8% after TKI combination, but there was no statistical difference in 5-year survival rate (16). At present, systemic therapy including ICIs, TKI and monoclonal antibody is also an important means for the treatment of advanced HCC. 50% to 60% of HCC patients will receive systemic therapy in the advanced stage and the overall survival rate and quality of life will be significantly improved (17). The combination of Atezolizumab (anti-PDL1 antibody) and bevacizumab (anti-VEGF antibody) has been reported to more than double the 8-month survival of advanced HCC (18). Ramucirumab has also been shown to improve survival time under monotherapy regiments for advanced HCC (19). Although there are currently no biomarkers to confirm that the population benefits from ICI monotherapy, clinical benefits have been demonstrated for 15-20% of HCC patients (20–23). Metuximab is a mouse monoclonal antibody fragment, HAb18F (ab’) 2, which can bind to HAbl8G/CD147, a highly expressed antigen on the surface of hepatocellular carcinoma cells, and block specific signal transduction to induce apoptosis of tumor cells. With the combination of the above two mechanisms, Licartin has dual antitumor effects, thus realizing a special tumor-targeted therapy model (24). In addition, it was found through the clinical application that repeated application of Licartin did not cause long-term irreversible injury to liver function and could be well tolerated (9, 25). Due to the specific binding of Metuximab to the tumor surface antigen, Licartin might theoretically bind more to HAbl8G/CD147 over-expressed on the hepatic carcinoma cells, while the binding rate of normal liver tissue to Licartin is low, thus reducing the influence on normal hepatocytes (26).

In the present study, there were 14 cases, accounting for 34.1%, in whom Licartin was distributed intensively in tumors. The analysis results suggested this was related to the previous history of the TACE operation. For patients with no previous history of TACE surgery, it could be better to select the tumor-supplying artery in the Licartin perfusion, so the Licartin might combine more completely with the tumor cells. After arterial embolization, the Licartin loss was reduced, and the 131-I aggregation in the normal liver tissue was reduced. Among the 13 patients in whom the Licartin distribution in the liver tumors was weakened compared with those in the normal tissues, 11 had a history of TACE surgery, accounting for 84.6%, and the other 2 patients had previous open surgery. In 13 cases, the super-selected tumor arteries were selected in 3 cases (23.3%). The proper hepatic artery and the common hepatic artery were selected in 5 cases (38.5%) each. In TACE surgery, the super-selected tumor arteries was the first choice, which was also a favorable factor to ensure that Licartin could better combine with liver tumors, and then the proper hepatic artery or common hepatic artery and other arteries will be selected. Sneiders reported that previous TACE surgery didn’t increase the risk of hepatic artery complications in patients with liver cancer undergoing liver transplantation (27). It indicated that TACE did not affect the main blood supply arteries of the liver, but changed the local blood supply and microenvironment of liver tumors. Previous TACE surgery embolized the main artery supplying the tumor. Although the blood supply to the tumor could be largely re-canalized after two weeks, it still could not reach the preoperative level. Therefore, Licartin perfused through both the common hepatic artery and the main artery of the intrinsic hepatic artery could not bind to the tumor ideally, resulting in a diminished distribution of Licartin in the tumor area. There was also a possibility that the previous TACE surgical treatment effectively caused ischemia necrosis or apoptosis of some liver tumors, which led to the failure of the later uptake of Licartin by this region and the reduction of local aggregation. Regardless of the cause, local liver biopsy was necessary to confirm. However, no matter which factor led to the reduced uptake of Licartin in liver tumors, it can not allow Licartin to exert its maximum therapeutic effect *in vivo*. Among the 14 patients with uniform distribution, 10 patients had previous open surgery only, 1 patient had previous open surgery and TACE, and 3 patients had previous TACE only. During Licartin perfusion, there were 8 cases with the super-selection of the tumor-supplying artery, 3 cases with the proper hepatic artery, and 3 cases with the common hepatic artery. The above indicates that previous open surgery did not affect the blood supply in recurrent tumors. Licartin perfused through the super-selective tumor-fed arteries could reach the entire liver through other channels or regenerated vascular channels. Therefore, these factors did not correlate with the present result of the distribution.

There were some limitations in this study. First, all patients with primary liver cancer treated with Licartin had not been treated as a single drug, so whether the combined chemotherapy drugs would affect the distribution of Licartin remained to be further studied. Besides, this result concluded from a single-center and small sample sized study remained to be proved by a multi-center and prospective study. Second, the potential molecular mechanism for the reduced uptake of tumor Licartin had not been studied. For example, some mutation factors led to insufficient expression or variation of habl8g/CD147 antigen on the surface of liver cancer cells, which made Licartin unable to recognize tumor cells. This need further research and analysis.

In summary, the main factor influencing the intrahepatic ^131^I distribution in Licartin combined with TACE for PHC was the history of previous TACE surgery, which might be related to the fact that previous TACE surgery would affect the tumor blood supply and thus lead to the inability of Licartin to bind adequately to tumor cells. Licatine treatment should be arranged in the first TACE operation. If TACE has been performed for many times in the past, the subsequent treatment with Licartin can not effectively reach the lesions that have been treated with TACE. Besides, age, previous open surgery history, the last interventional surgery, and the interval between treatment with Licatine did not significantly affect the distribution of Licatine. We hope our study can provide reference for a multicenter study to analyze whether these three different distributions of riccatine will affect the prognosis and survival rate of patients in the future.

## Data availability statement

The original contributions presented in the study are included in the article/supplementary material. Further inquiries can be directed to the corresponding author.

## Ethics statement

The studies involving human participants were reviewed and approved by Ethics Committee of Yunnan Cancer Hospital (KYLX2022065). The patients/participants provided their written informed consent to participate in this study.

## Author contributions

Conception and design of the research: P-JL. Acquisition of data: JY-L, MT. Analysis and interpretation of the data: JL, J-LZ. Statistical analysis: F-KC. Writing of the manuscript: MT. Critical revision of the manuscript for intellectual content: P-JL. All authors contributed to the article and approved the submitted version.
